# Notch-Mediated Generation of Monocyte-Derived Langerhans Cells: Phenotype and Function

**DOI:** 10.1016/j.jid.2020.05.098

**Published:** 2021-01

**Authors:** Lydia Bellmann, Claudia Zelle-Rieser, Paul Milne, Anastasia Resteu, Christoph H. Tripp, Natascha Hermann-Kleiter, Viktoria Zaderer, Doris Wilflingseder, Paul Hörtnagl, Maria Theochari, Jessica Schulze, Mareike Rentzsch, Barbara Del Frari, Matthew Collin, Christoph Rademacher, Nikolaus Romani, Patrizia Stoitzner

**Affiliations:** 1 Department of Dermatology, Venereology & Allergology, Medical University of Innsbruck, Innsbruck, Austria; 2 Translational and Clinical Research Institute, Faculty of Medical Sciences, Newcastle University, Newcastle upon Tyne, United Kingdom; 3 Institute of Cell Genetics, Department for Genetics and Pharmacology, Medical University of Innsbruck, Innsbruck, Austria; 4 Institute of Hygiene and Medical Microbiology, Medical University of Innsbruck, Innsbruck, Austria; 5 Central Institute for Blood Transfusion and Immunological Department, Medical University of Innsbruck, Innsbruck, Austria; 6 Department of Biomolecular Systems, Max Planck Institute of Colloids and Interfaces, Potsdam, Germany; 7 Department of Plastic, Reconstructive and Aesthetic Surgery, Medical University of Innsbruck, Innsbruck, Austria

**Keywords:** A647, AlexaFluor-647, DC, dendritic cell, LC, Langerhans cell, MHC, major histocompatibility complex, MLR, mixed leukocyte reaction, moLC, monocyte-derived LC, PolyI:C, polyinosinic:polycytidylic acid, RNA-seq, RNA sequencing, Th, T helper, TLR, toll-like receptor

## Abstract

Langerhans cells (LCs) in the skin are a first line of defense against pathogens but also play an essential role in skin homeostasis. Their exclusive expression of the C-type lectin receptor Langerin makes them prominent candidates for immunotherapy. For vaccine testing, an easily accessible cell platform would be desirable as an alternative to the time-consuming purification of LCs from human skin. Here, we present such a model and demonstrate that monocytes in the presence of GM-CSF, TGF-β1, and the Notch ligand DLL4 differentiate within 3 days into CD1a^+^Langerin^+^cells containing Birbeck granules. RNA sequencing of these monocyte-derived LCs (moLCs) confirmed gene expression of LC-related molecules, pattern recognition receptors, and enhanced expression of genes involved in the antigen-presenting machinery. On the protein level, moLCs showed low expression of costimulatory molecules but prominent expression of C-type lectin receptors. MoLCs can be matured, secrete IL-12p70 and TNF-α, and stimulate proliferation and cytokine production in allogeneic CD4^+^ and CD8^+^ T cells. In regard to vaccine testing, a recently characterized glycomimetic Langerin ligand conjugated to liposomes demonstrated specific and fast internalization into moLCs. Hence, these short-term in vitro‒generated moLCs represent an interesting tool to screen LC-based vaccines in the future.

## Introduction

Langerhans cells (LCs) are specialized antigen-presenting cells residing in the epidermis of the skin in close contact with neighboring keratinocytes (Romani et al., 2010a). They are also found in mucosal, vaginal, and other epithelia (Hovav, 2018). LCs express the C-type lectin receptor Langerin, which induces the formation of Birbeck granules and other typical dendritic cell (DC) markers such as major histocompatibility complex (MHC) class II molecules, CD11c, CD1a, and CD1c (Valladeau et al., 2000). During embryonic development, LCs arise from yolk sac macrophages and fetal liver monocytes and later restore their numbers by local self-renewal in the absence of inflammation (Merad et al., 2002).

LC homeostasis crucially depends on the cytokine TGF-β that is produced by keratinocytes and LCs, acting in an autocrine feedback loop (Borkowski et al., 1996, Kaplan et al., 2007). Furthermore, BMP7 is also involved in LC differentiation, especially expressed in the prenatal but also adult basal epidermal layers (Yasmin et al., 2013). In steady state, LCs migrate to the lymph node where they present self-antigens to T cells contributing to immune tolerance. Upon inflammation or pathogen invasion, LCs are activated, migrate to the lymph node, and interact with various T cells (Clausen and Stoitzner, 2015, Romani et al., 2010a, West and Bennett, 2017). Strong inflammation leaves an empty niche that is seeded with blood monocytes in a CCR2-dependent manner that are capable of differentiating into a transient LC pool. After 6–8 weeks, a lasting not-yet-identified myeloid precursor with self-renewing potential and high Langerin expression seeds the epidermis (Collin and Milne, 2016, Ginhoux et al., 2006, Seré et al., 2012). A recent study reported that monocytes could give rise to long-lived LCs in the murine epidermis upon inflammation besides representing the progenitor of short-lived LCs (Ferrer et al., 2019). Not only do LCs initiate priming in the lymph node but they also have important skin-resident functions such as the interaction with regulatory T cells, resident memory T cells, and NK cells (Ortner et al., 2017, West and Bennett, 2017).

In vitro, it was shown that CD14^+^ monocytes can be differentiated into Langerin-expressing cells when cultured with GM-CSF, IL-4, and TGF-β (Geissmann et al., 1998, Guironnet et al., 2002, Picarda et al., 2016). CD1c^+^ blood DCs and CD34^+^ progenitor cells share this feature when cultured with GM-CSF and BMP7 (Milne et al., 2015, Yasmin et al., 2013) or with thymic stromal lymphopoietin and TGF-β (Martínez-Cingolani et al., 2014). Furthermore, upon Notch ligation with the immobilized Notch ligand DLL1, monocytes acquired an LC phenotype with high Langerin expression (Hoshino et al., 2005). Culturing monocytes on OP9 stromal cells transduced to express DLL1 or DLL4 (OP9-DLL1 or OP9-DLL4) together with GM-CSF and TGF-β also induced high Langerin expression within 3 days (Milne et al., 2017). This latter method allowed rapid and very efficient differentiation of monocyte-derived LCs (moLCs). However, their properties, foremost functional proficiency, have not been studied so far. We, therefore, investigated here in detail the functional characteristics of Langerin-expressing cells derived from human monocytes with the Notch ligand DLL4. Indeed, we demonstrate that they have many LC features, stimulate T-cell proliferation and cytokine secretion, and can be loaded with Langerin-targeted vaccines. Thus, these in vitro‒generated moLCs can be a useful model to study LC function and to test future LC-based immunotherapies.

## Results

### Notch ligation allows the rapid generation of moLCs with the expression of Langerin and Birbeck granules

**Figure 1:**
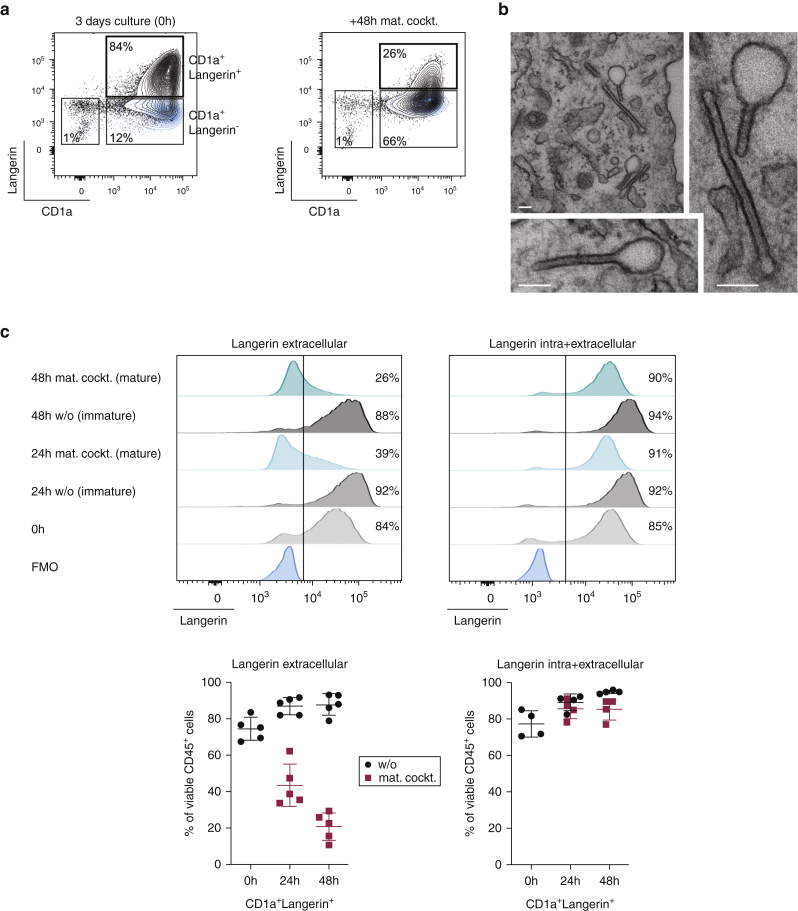
**Notch ligation allows rapid differentiation of moLCs with high expression of Langerin and Birbeck granules.** (**a**) Representative density plots showing surface Langerin and CD1a after 3-day culture of monocytes on OP9-DLL4 with TGF-β1 and GM-CSF. Mat. cockt. was added for an additional 48 hours, and cells were analyzed. (**b**) Representative electron microscopy of Birbeck granules from two moLCs. The right panel was enlarged from the top panel. Bars = 100 nm. (**c**) Representative histograms for Langerin surface (left, extracellular) and Langerin total (right, intra and extracellular) are shown on moLCs at different times before (0 h) and after maturation (mat. cockt.) or medium control (w/o). Summary graphs for five experiments show percentages of CD1a^+^Langerin^+^ cells within viable CD45^+^ cells. Mean ± SD. FMO, fluorescence minus one; h, hour; LC, Langerhans cell; mat. cockt, maturation cocktail; moLC, monocyte-derived LC; w/o, without.

**Supplementary Figure S1:**
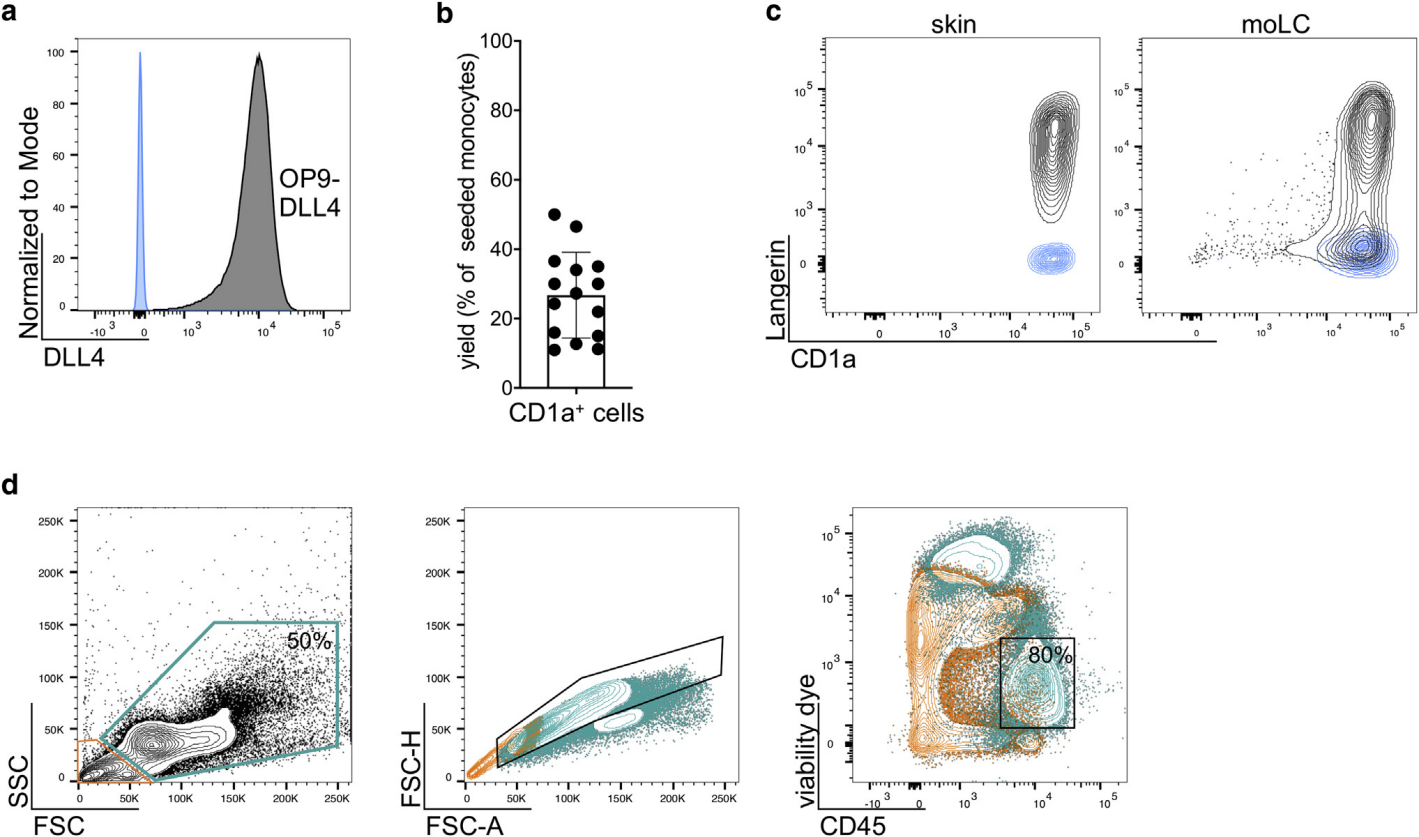
**Characterization of moLC culture.** (**a**) Stromal OP9-DLL4 cells were analyzed for their expression of DLL4 by flow cytometry. Isotype-control mAb is displayed in blue. (**b**) Cell yields from moLC cultures were determined by calculating the percentages of harvested cells from initially seeded monocytes. (**c**) Langerin and CD1a expression on LCs isolated from human skin (left) and moLCs (right). Isotype-control mAb is displayed in blue. (**d**) Gating strategy after 3 days of culture of monocytes on OP9-DLL4 plus TGF-β1 and GM-CSF. Detached FSC^low^ OP9-DLL4 cells are mostly found in the orange part, with 80% being dead; FSC^high^ OP9-DLL4 cells are found together with the in vitro‒generated cells in the petrol blue part but can be excluded by their lack of CD45. FSC, forward scatter; LC, Langerhans cell; moLC, monocyte-derived LC; SSC, side scatter.

To generate moLCs, blood CD14^+^ monocytes were seeded on a monolayer of OP9-DLL4 stromal cells (Supplementary Figure S1a) and were cultured in the presence of TGF-β1 and GM-CSF for 3 days (Milne et al., 2017). Approximately 30% of initially plated monocytes were able to differentiate into CD1a-expressing cells (Supplementary Figure S1b). The vast majority (74.5% ± 6.3%) coexpressed Langerin (Figure 1a and c), phenotypically similar to CD1a^+^Langerin^+^ LCs isolated from human skin (Supplementary Figure S1c). A small percentage of CD1a^+^Langerin^−^ cells (13.2% ± 3.3%) was also present under these culture conditions (Figure 1a and Supplementary Figure S3a). The monolayer of OP9-DLL4 cells detached when moLCs were collected; however, these stromal cells could be excluded from subsequent analysis by their lack of CD45 (Supplementary Figure S1d). Electron microscopy of the in vitro‒generated moLCs proved the presence of unequivocal Birbeck granules (Figure 1b), a hallmark of LCs in the epidermis (Romani et al., 2010a, Wolff, 1967). They appeared in their characteristic rod and tennis racket shapes with the striated central line (Figure 1b), a defining feature, and occurred in about one-third of cell section profiles. Importantly, the in vitro‒generated moLCs matured in response to the standard DC-cytokine maturation cocktail containing IL-1β, IL-6, TNF-α, and prostaglandin E2 (Jonuleit et al., 1997) within 24–48 hours. Surface Langerin was internalized during the maturation process, but Langerin molecules could still be detected intracellularly (Figure 1a and c). Thus, the percentages of CD1a^+^Langerin^+^ cells within the pool of viable CD45^+^ cells did not change upon maturation; however, for their detection, Langerin staining had to be performed on permeabilized cells (Figure 1c).

We conclude that monocytes, in the presence of the Notch ligand DLL4, TGF-β1, and GM-CSF, differentiate rapidly to moLCs in vitro and resemble skin LCs in regard to CD1a and Langerin expression and presence of Birbeck granules. Therefore, in vitro‒differentiated moLCs could be an attractive alternative to the LCs isolated from the skin*.*

### In vitro‒generated moLCs express LC-related molecules and respond to toll-like receptor/ RIG-I-like receptor ligands

**Figure 2:**
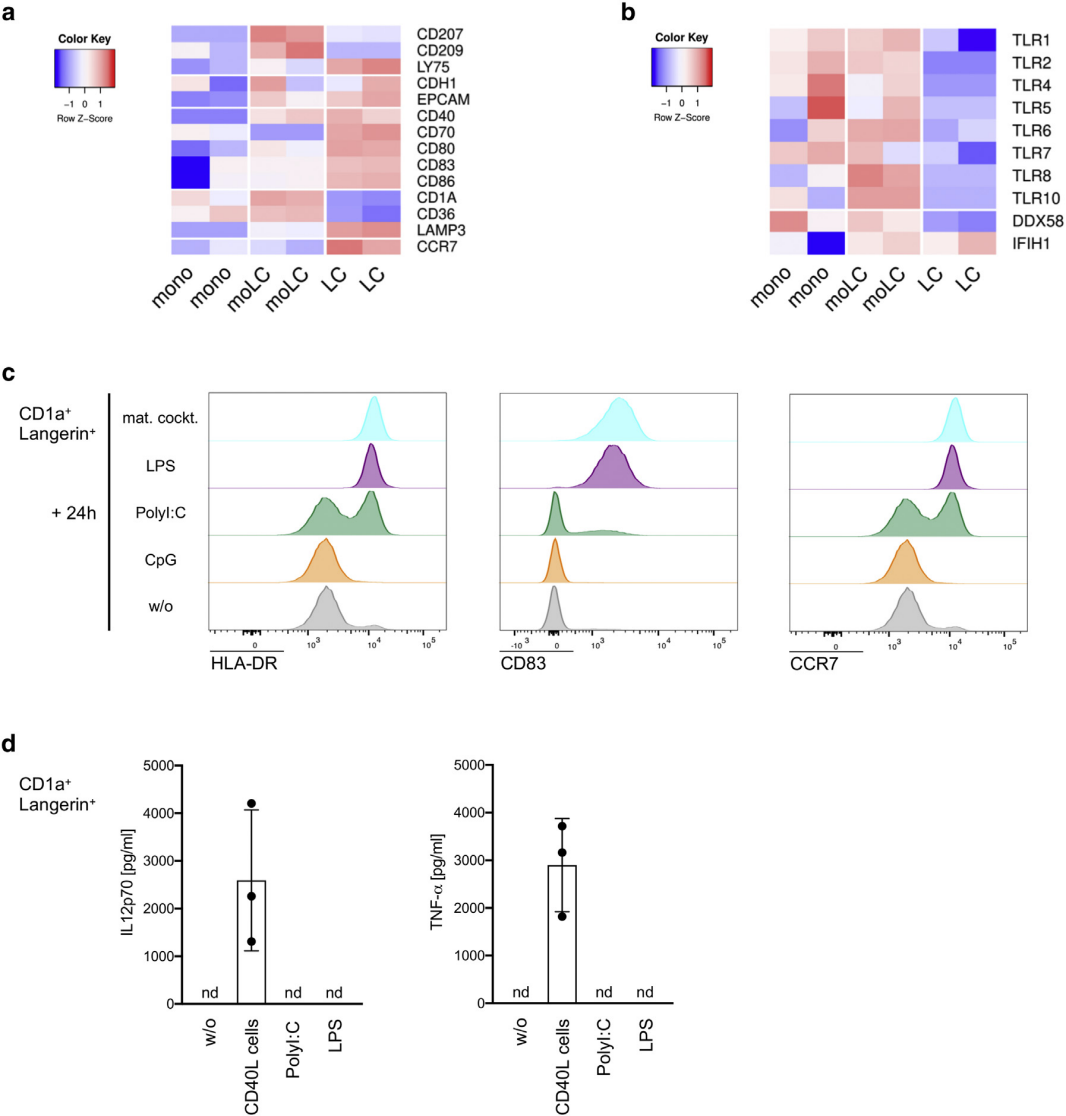
**In vitro‒generated moLCs express LC-related molecules and respond to TLR or RLR ligands.** (**a, b**) Sorted monocytes, moLCs, and migratory skin LCs from two different donors were analyzed by RNA-seq. Heatmap depicts the normalized and relative expression (z score) of (**a**) LC-related genes and (**b**) TLR and RLR genes. (**c**) MoLCs were analyzed by flow cytometry after 24 hours with a cytokine mat. cockt, LPS, PolyI:C, or CpG for the expression of the maturation markers HLA-DR and CD83 and the chemokine receptor CCR7. Representative histograms of one donor (n = 2–3) are shown. (**d**) A total of 100,000 moLCs were cultured with 50,000 CD40L cells or TLR ligands for 24 hours, and IL-12p70 as well as TNF-α were measured in supernatants by ELISA. Mean ± SD, n = 2–3. h, hour; LC, Langerhans cell; LPS, lipopolysaccharide; mat.cockt, maturation cocktail; moLC, monocyte-derived LC; nd, not detectable; PolyI:C, polyinosinic:polycytidylic acid; RLR, RIG-I‒like receptor; TLR, toll-like receptor; RNA-seq, RNA sequencing; w/o, without.

**Supplementary Figure S2:**
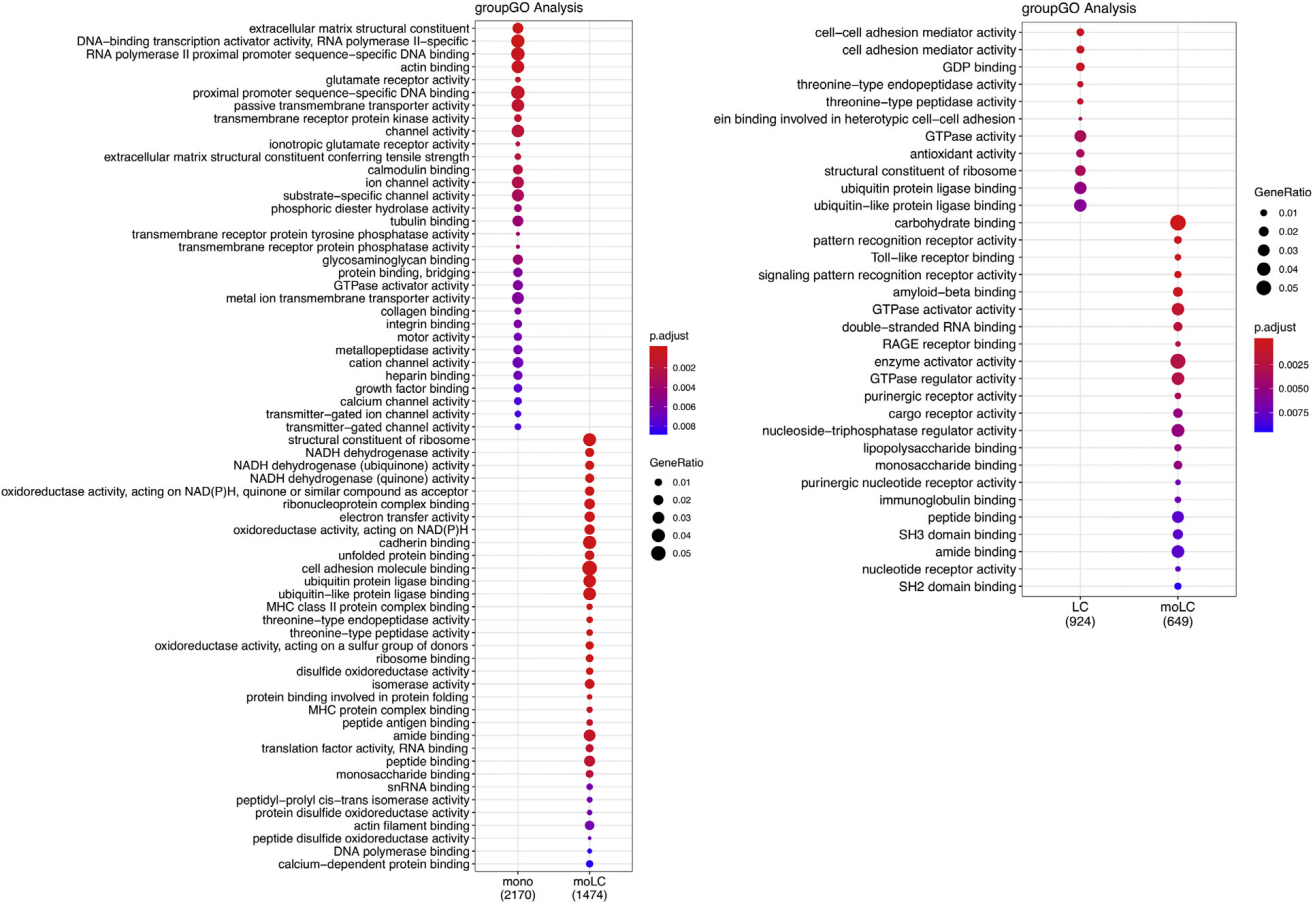
**GO analysis of moLCs.** Gene expression from sorted CD14^+^ monocytes, CD1a^+^Langerin^+^ moLCs, and CD1a^+^Langerin^+^ migratory skin LCs from two different donors were analyzed by RNA-seq. GO analysis was performed on differentially expressed genes with higher expression in monocytes versus moLCs or skin LCs versus moLCs. All differentially regulated pathways (adjusted *P*-value < 0.01) are listed. GO, gene ontology; LC, Langerhans cell; moLC, monocyte-derived LC; RNA-seq, RNA sequencing.

We performed RNA sequencing (RNA-seq) to investigate the differential expression of genes among sorted CD1a^+^Langerin^+^ moLCs, CD14^+^ monocytes, and skin LCs emigrated from the epidermis. Gene ontology analysis of significantly enriched terms demonstrated pronounced differences in moLCs compared to monocytes, for example, peptide antigen binding and differences in moLCs compared to skin LCs, for example, toll-like receptor (TLR) binding and carbohydrate binding (Supplementary Figure S2). A closer look at the gene expression of LC-related molecules confirmed that moLCs strongly upregulated genes for cell adhesion (*EPCAM* and *E-cadherin* [*CDH1*]), for antigen uptake and processing (*C-type lectin receptors*, *CD36*, *LAMP3*), and for T-cell costimulation (*CD80*, *CD83*) in comparison with monocytes (Figure 2a). Analysis of pattern recognition receptor genes revealed the mRNA expression of *TLR1*, *2, 4–8, 10, RIG-I* (*DDX58*), and *MDA-5* (*IFIH1*) in moLCs and a lack of *TLR3* and *TLR9* (Figure 2b). Moreover, moLCs displayed an immature phenotype, whereas migratory skin LCs upregulated genes for maturation markers, for example, *CD83* and *CCR7* (Figure 2a). Stimulation of CD1a^+^Langerin^+^ moLCs with different TLR or RIG-I‒like receptor ligands resulted in an increased expression of the maturation markers HLA-DR and CD83 and the chemokine receptor CCR7 after a 24-hour culture with polyinosinic:polycytidylic acid (PolyI:C) (TLR3, RIG-I, MDA-5), lipopolysaccharide (TLR4), and the maturation cocktail but not with CpG (TLR9) according to the RNA expression pattern of these receptors in moLCs (Figure 2c). Furthermore, CD1a^+^Langerin^+^ moLCs secreted IL-12p70 and TNF-α after culturing with cells expressing the human CD40L (CD40L cells), mimicking the interaction with T cells (Figure 2d). TLR agonists alone did not induce IL-12p70 and TNF-α secretion (Figure 2d).

Thus, moLCs express typical LC markers and pattern recognition receptors that allow them to respond to TLR agonists and produce T helper (Th)1-inducing cytokines after additional CD40 ligation.

### In vitro‒generated moLCs express maturation markers and C-type lectin receptors

**Figure 3:**
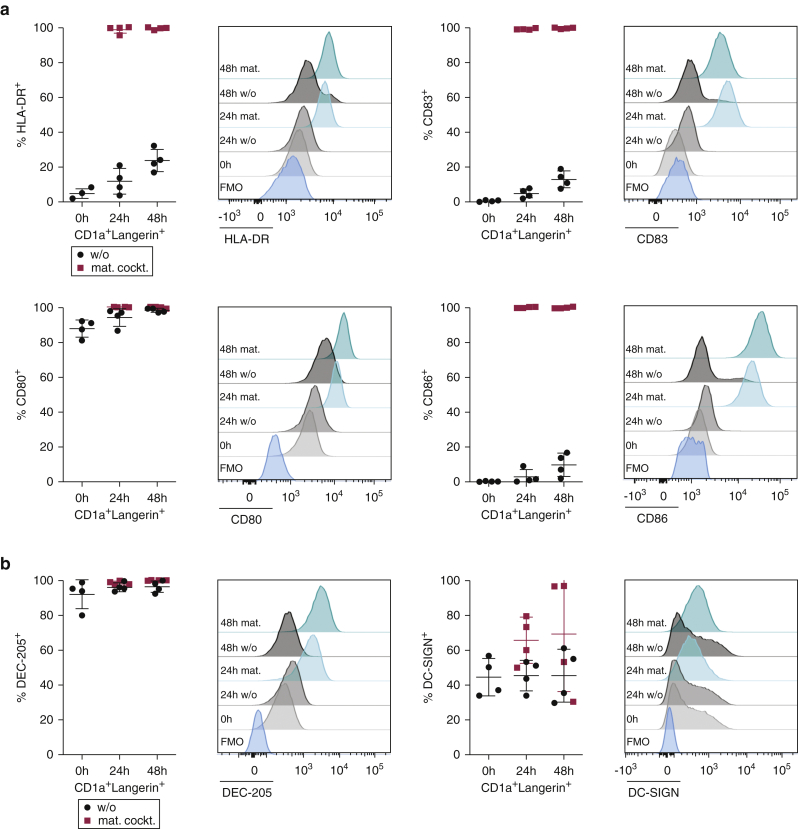
**In vitro‒generated moLCs express maturation markers and C-type lectin receptors DEC-205 and DC-SIGN.** (**a, b**) In vitro‒generated moLCs (CD1a^+^Langerin^+^) were analyzed by flow cytometry after 3 days of culturing (0 h) or after 24 h or 48 h in the presence (mat.cockt.) or absence (w/o) of a cytokine mat. cockt. for the expression of (**a**) the maturation markers HLA-DR, CD83, CD80, and CD86 and (**b**) C-type lectin receptors DEC-205 and DC-SIGN. Summary graphs for four experiments show percentages of receptor-positive cells within CD1a^+^Langerin^+^ cells. Mean ± SD. DC, dendritic cell; FMO, fluorescence minus one; h, hour; LC, Langerhans cell; mat. cockt, maturation cocktail; moLC, monocyte-derived LC; w/o, without.

**Supplementary Figure S3:**
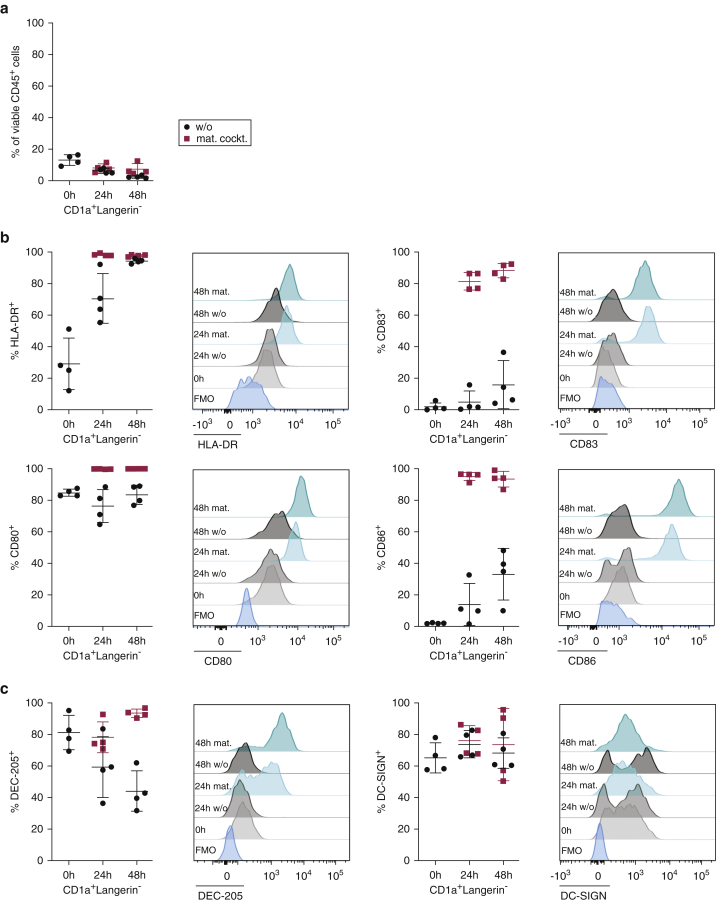
**Characterization of the minor subset of CD1a^+^Langerin^−^ cells.** (**a–c**) CD1a^+^Langerin^−^ cells were analyzed by flow cytometry after 3-day culture (0 hour) or after 24 h or 48 h in the presence (mat. cockt.) or absence (w/o) of a cytokine mat. cockt. (**a**) Percentages of CD1a^+^Langerin^−^ cells within viable CD45^+^ cells and the expression of (**b**) the maturation markers HLA-DR, CD83, CD80, and CD86 and (**c**) the C-type lectin receptors DEC-205 and DC-SIGN are shown. Summary graphs for four experiments are shown; mean ± SD. FMO, fluorescence minus one; h, hour; mat.cockt, maturation cocktail; w/o, without.

On the protein level, we observed that moLCs were in an immature stage with low HLA-DR, CD83, and CD86 expression on their surface (Figure 3a). The expression of all of these markers increased within 24–48 hours when moLCs were cultured with the DC-cytokine maturation cocktail. Interestingly, about 85% of CD1a^+^Langerin^+^ cells were positive for CD80 before any maturation stimulus; nevertheless, the geometric mean fluorescence intensity of CD80 increased with the cytokine cocktail (Figure 3a). The minor population of CD1a^+^Langerin^−^ cells also showed upregulation of the different maturation markers upon culturing with the cytokine cocktail (Supplementary Figure S3b). In line with the RNA-seq data, most of the immature moLCs expressed DEC-205, and its expression increased on mature moLCs (Figure 3b). A similar pattern was seen in the minor population of CD1a^+^Langerin^−^ cells (Supplementary Figure S3c). DC-SIGN expression was more heterogeneous between the different experiments (35–65% of moLCs) but was also partly upregulated during maturation (Figure 3b and Supplementary Figure S3c).

In summary, in vitro‒generated moLCs resemble immature LCs as determined on the mRNA and protein level. Upon maturation, moLCs strongly upregulated HLA-DR, CD83, and CD86. Furthermore, besides the C-type lectin receptor Langerin, they also express DEC-205 and DC-SIGN on their surface.

### In vitro‒generated mature moLCs stimulate allogeneic T cells and induce a Th1/Th17 cytokine pattern

**Figure 4:**
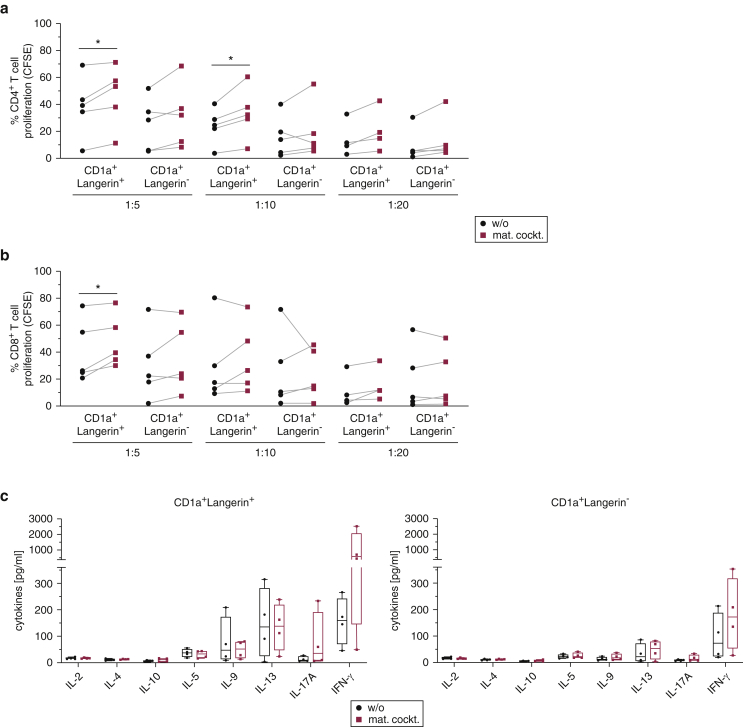
**In vitro‒generated mature moLCs stimulate allogeneic T cells and induce higher Th1/Th17 cytokines.** (**a–c**) In vitro‒generated CD1a^+^Langerin^+^ moLCs and CD1a^+^Langerin^−^ cells were sorted, cultured for 24 hours in the presence (mat. cockt.) or absence (w/o) of a cytokine mat. cockt. and then cocultured in different ratios with CFSE-labeled allogeneic PBLs. After 5 days, CFSE dilution was analyzed by flow cytometry for (**a**) CD4^+^ T cells and (**b**) CD8^+^ T cells for the indicated ratios. (**c**) Supernatants were collected on day 5 of coculture, and secretion of T cell cytokines for the 1:10 ratio was analyzed by ProcartaPlex Immunoassay. Summary graphs for four experiments are displayed as box plots with individual blood donors. CFSE, carboxyfluorescein succinimidyl ester; LC, Langerhans cell; mat. cockt, maturation cocktail; moLC, monocyte-derived LC; PBL, peripheral blood lymphocyte; Th, T helper; w/o, without.

**Supplementary Figure S4:**
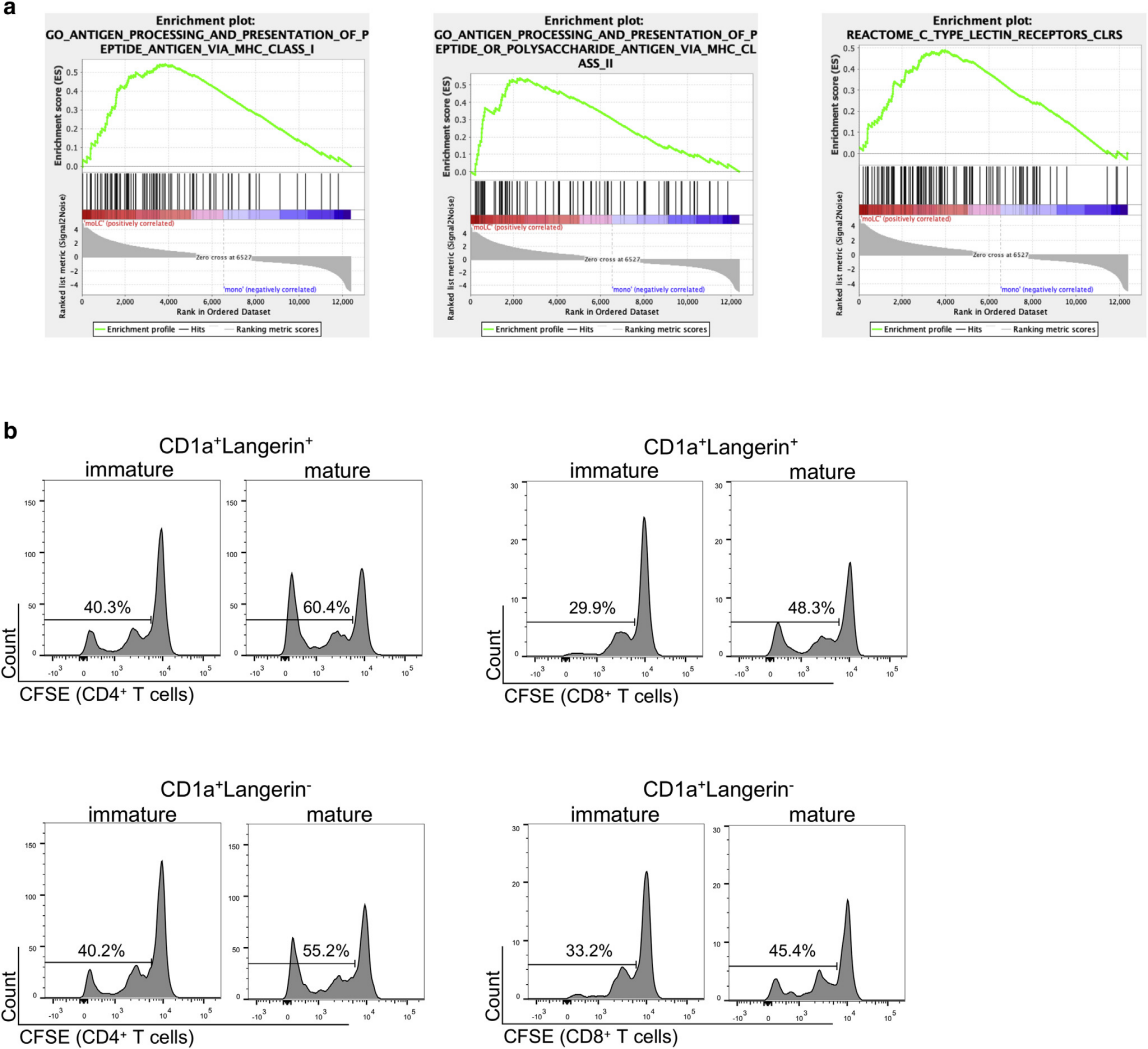
**Functional aspects of moLCs.** (**a**) Enrichment plots show GO terms for antigen presentation in the context of MHC I (GO:0002474) and MHC II (GO:0002504) as well as the Reactome for C-type lectin receptor pathway (R-HSA-5621481). Data are accessible through GSE141048. (**b**) CD1a^+^Langerin^+^ moLCs and CD1a^+^Langerin^−^ cells were sorted, cultured for 24 h in the presence or absence of a cytokine mat. cockt. and then cocultured in different ratios with CFSE-labeled allogeneic PBLs. After 5 days, CFSE dilution was analyzed by flow cytometry for CD4^+^ T cells and CD8^+^ T cells. Representative histograms are for CFSE dilution for 1:10 ratio. CFSE, carboxyfluorescein succinimidyl ester; GO, gene ontology; h, hour; LC, Langerhans cell; mat. cockt., maturation cocktail; MHC, major histocompatibility complex; moLC, monocyte-derived LC; PBL, peripheral blood lymphocyte.

Geneset enrichment analysis revealed that moLCs had significantly higher antigen processing and presentation capabilities through MHC class I and II molecules and enrichment of Reactome C-type lectin receptor pathway than monocytes (Supplementary Figure S4a). Thus, we determined the T cell stimulatory capacity of the in vitro‒generated moLCs in allogeneic mixed leukocyte reactions (MLRs). We sorted CD1a^+^Langerin^+^ and CD1a^+^Langerin^−^ cells and cultured them for 24 hours in the presence or absence of the DC-cytokine maturation cocktail. Both cell types were cocultured with carboxyfluorescein succinimidyl ester‒labeled allogeneic peripheral blood lymphocytes at the ratios of 1:5, 1:10, and 1:20 for 5 days. Mature moLCs showed a higher capacity in stimulating allogeneic CD4^+^ as well as CD8^+^ T cells than immature ones as determined by carboxyfluorescein succinimidyl ester dilution (Figure 4a and b and Supplementary Figure S4b). The minor population of CD1a^+^Langerin^−^ cells was not grossly different in their potency to induce CD4^+^ and CD8^+^ T cell proliferation when compared with moLCs (Figure 4a and b and Supplementary Figure S4b).

To investigate the potential of moLCs to induce a specific CD4 helper subset response, the supernatants from the MLRs were collected and analyzed with a ProcartaPlex Immunoassay to measure T cell cytokines. Albeit detectable**,** IL-2, IL-4, and IL-10 cytokine levels were very low. Immature moLCs induced a mixed Th1, Th2, and Th9 response, whereas mature moLCs increased the T cell secretion of IL-17A and IFN-γ (Figure 4c). All cytokines were secreted at very low levels by T cells cocultured with the CD1a^+^Langerin^−^ cells; however, a trend toward more IFN-γ was still detected in cocultures with mature than immature CD1a^+^Langerin^−^ cells (Figure 4c).

To conclude, the in vitro‒generated moLCs are able to stimulate effectively allogeneic T-cell proliferation and cytokine secretion, especially when matured with the DC-cytokine cocktail. Moreover, moLCs induced a mixed T cell cytokine secretion signature with increased IFN-γ and IL-17A after maturation.

### In vitro‒generated moLCs can be targeted with a glycomimetic Langerin ligand

**Figure 5:**
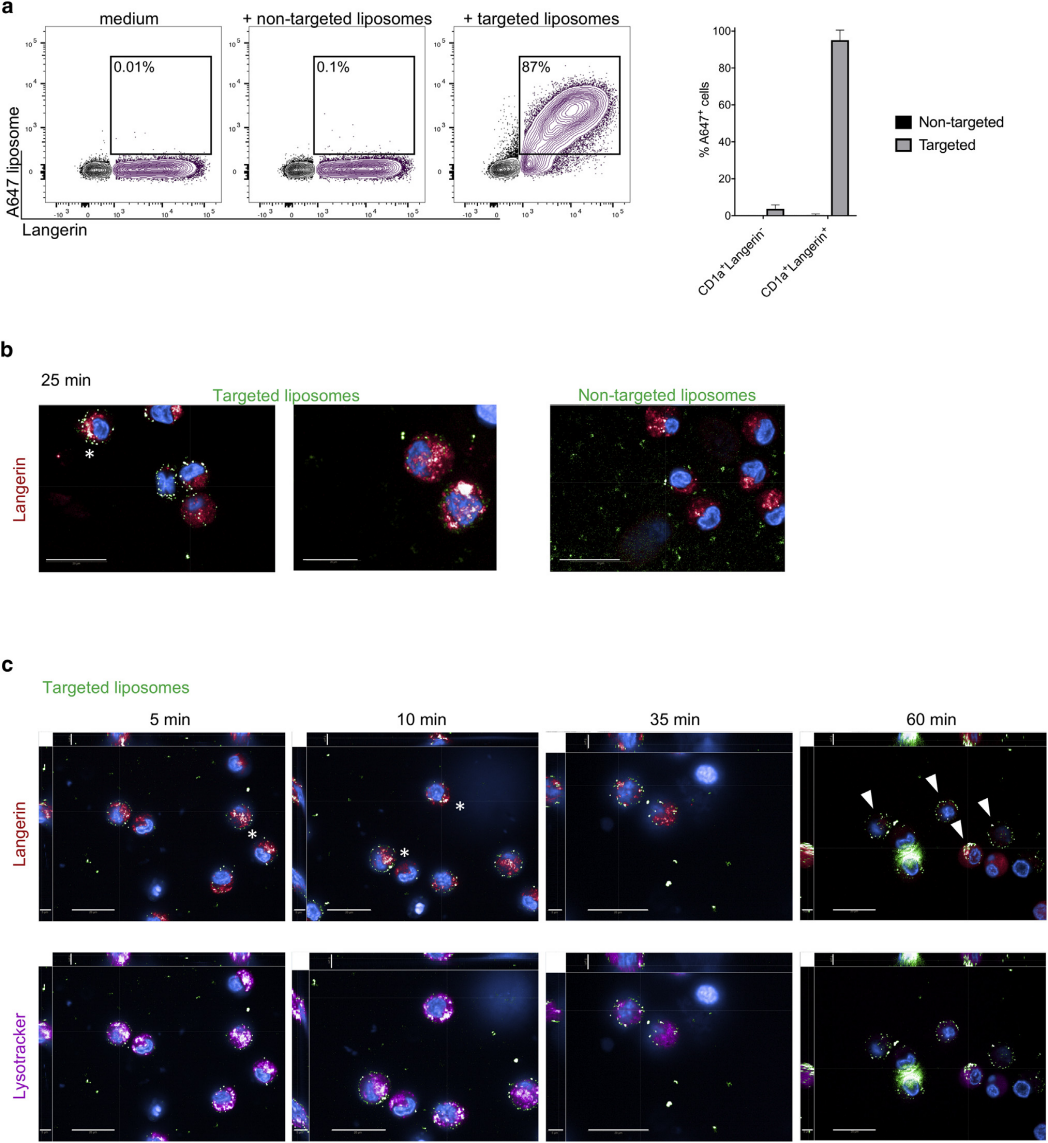
**MoLCs can be targeted with a glycomimetic Langerin ligand.** (**a–c**) In vitro‒generated moLCs and CD1a^+^Langerin^−^ cells were incubated with A647-labeled liposomes coated with a Langerin ligand (targeted liposomes) or nontargeted liposomes. (**a**) Internalization after 1 hour at 37 °C was determined by positive A647 signal by flow cytometry. Summary graph for four experiments is shown; Mean ± SD. (**b, c**) Binding and internalization were followed by Operetta High-Content Imaging System for (**b**) 25 minutes or (**c**) the indicated time periods. Liposomes are displayed in green, Langerin in red, and Lysotracker in pink. White asterisks indicate colocalization of liposomes with Langerin, and white arrows show internalized liposomes. Bars = 20 μm. Left and upper part of each picture show xyz-stack side view (scale bars = 5 μm). A647, AlexaFluor-647; LC, Langerhans cell; moLC, monocyte-derived LC.

We recently described the specific binding and uptake of liposomes coated with a recently developed glycomimetic Langerin ligand to human LCs (Wamhoff et al., 2019). This vaccine platform is of particular interest in skin vaccination approaches because any antigen or drug can be encapsulated into liposomes for targeted delivery to LCs. Because the access to human skin can be difficult and isolation of LCs can be very work intensive, we assessed the potential of the in vitro‒generated moLCs to be used as a model for purified LCs. We incubated moLC cultures for 1 hour with Langerin ligand‒coated liposomes and confirmed that targeted liposomes were specifically internalized into Langerin^+^ moLCs and not into Langerin^−^ cells (Figure 5a). Furthermore, we visualized the uptake of the targeted liposomes by Operetta High-Content Imaging System for different time points in a mixed culture of moLC and CD1a^+^Langerin^−^ cells. Targeted liposomes bound to the cell surface of moLCs within 5–10 minutes and colocalized with Langerin in contrast to nontargeted liposomes (Figure 5b and c). In a time course, we observed that within 40 minutes, liposomes were internalized and colocalized with lysotracker, a marker to visualize the lysosomal compartment (Figure 5c).

Taken together, in vitro‒generated Langerin-expressing moLCs can serve as a convenient cellular platform to test different immunotherapeutic approaches, for example, LC-targeting liposomes.

## Discussion

LCs reside in the epidermis where they survey the tissue for invading pathogens (Romani et al., 2010a). Owing to their specific surface receptor expression and superficial localization, they are promising targets for immunotherapy through intradermal and transcutaneous administration routes (Romani et al., 2010b, Stoitzner et al., 2010). Working with LCs can be challenging because there are limitations in regard to access to fresh human skin, and isolation procedures are work intensive. Therefore, methods were developed to generate LC-like cells from CD14^+^ monocytes, CD1c^+^ blood DCs, or CD34^+^ progenitor cells (Geissmann et al., 1998, Hoshino et al., 2005, Milne et al., 2017, Milne et al., 2015, Strobl et al., 1997).

Notch ligation is an important factor to induce high Langerin expression in vitro (Hoshino et al., 2005, Milne et al., 2017). In the 3-day protocol used here, the Notch ligand DLL4 together with GM-CSF and TGF-β induced high Langerin and CD1a expression as well as Birbeck granules, all hallmarks of immature LCs residing in the epidermis. Langerin is a C-type lectin receptor that is internalized upon activation and is responsible for the formation of Birbeck granules (Valladeau et al., 2000), a recycling endosomal compartment for Langerin that allows receptor trafficking to the cell surface of LCs (Mc Dermott et al., 2002). We confirm and extend the few reports on the induction of Birbeck granules and Langerin in moLCs (Geissmann et al., 1998, Guironnet et al., 2002, Picarda et al., 2016). Our method yields well-developed Birbeck granules virtually indistinguishable from the structures in skin LCs. Another interesting finding was that the moLCs differentiated by Notch ligation quickly internalized Langerin upon maturation, a feature reported also for mature human epidermal LCs (Valladeau et al., 1999). Moreover, on mRNA level, we also detected *EPCAM* expression, an adhesion molecule required by skin LCs for tissue residency (Romani et al., 2010a). Thus, the method described here has several advantages over other methods that used GM-CSF and TGF-β alone (Geissmann et al., 1998, Guironnet et al., 2002, Picarda et al., 2016) or in combination with IL-15 (Mohamadzadeh et al., 2001), IFN-α (Maarifi et al., 2020), or BMP7 (Yasmin et al., 2013) because Notch ligation allows differentiation of moLCs within 3 days with consistent, high levels of Langerin and formation of Birbeck granules.

Besides Langerin, moLCs also expressed other C-type lectin receptors such as DEC-205 and DC-SIGN on the mRNA and protein level, which were upregulated during maturation. These C-type lectin receptors are differentially expressed in the skin: DEC-205 is upregulated with maturation on all skin DC subtypes including LCs (Ebner et al., 2004, Stoitzner et al., 2014), whereas DC-SIGN is only found on dermal CD14^+^ myeloid cells (Ebner et al., 2004, McGovern et al., 2014). DC-SIGN expression on moLCs, although very heterogeneous, hints at the provenance from monocytes and implies that moLCs might still express some monocytic markers.

With a defined cytokine cocktail, moLCs matured and displayed high levels of HLA-DR, CD80, CD83, and CD86 on their surfaces. Similarly, moLCs were responsive to TLR or RIG-I‒like receptor ligands according to their gene expression profile and upregulated HLA-DR, CD83, and CCR7 after stimulation with lipopolysaccharide or PolyI:C but not with CpG. Because *TLR3* mRNA was undetectable in the RNA-seq analysis, we confirmed the mRNA expression of the RIG-I‒like receptors *RIG-I* and *MDA-5* in moLCs as alternative receptors for PolyI:C. Accordingly, it was reported recently that MDA-5 is the main sensor for PolyI:C in skin LCs (Tajpara et al., 2018). This is of potential clinical importance because PolyI:C is already being used as an adjuvant in DC-based cancer vaccines (Dhodapkar et al., 2014). TLR or RIG-I‒like receptor agonists alone were unable to induce cytokine secretion by moLCs, whereas CD40 ligation was required for the induction of IL-12p70 and TNF-α. These results demonstrate that moLCs can be matured, comparable to skin LCs (Romani et al., 1989), but might be superior in regards to cytokine production because skin LCs are low producers of IL-12p70 (Ebner et al., 2007, Ebner et al., 2001, Peiser et al., 2004, Ratzinger et al., 2004).

Functionally, mature moLCs had a higher allogeneic CD4^+^ and CD8^+^ T cell stimulation capacity than immature moLCs. This mirrors the situation with human skin LCs that were shown to be weak allogeneic T cell stimulators when freshly isolated from the epidermis (Braathen and Thorsby, 1980) but potently stimulated T cells when cultured for 3–5 days (Romani et al., 1989). Expression of the costimulatory molecule CD80 on immature moLCs most likely contributes to their allogeneic stimulation potential. The cytokines secreted by T cells showed a mixed Th1/Th2 and Th9 picture when stimulated with immature moLCs, whereas mature moLCs enhanced IFN-γ and IL-17A secretion. This is in accordance with observations that in vitro‒generated and matured LCs induced IFN-γ‒producing T cells and IL-17 secretion (Gramlich et al., 2019, Hoshino et al., 2005). Furthermore, LCs either isolated from human skin or differentiated in vitro were able to induce the differentiation of CD4^+^ T cells into Th2 and Th1 cells (Furio et al., 2010, Hoshino et al., 2005, Martínez-Cingolani et al., 2014). Similarly, LCs obtained from human skin induced Th2 and Th17 cells and cross-primed CD8^+^ T cells (Klechevsky et al., 2008, Mathers et al., 2009). The RNA-seq analysis hinted at enhanced gene expression of antigen-presentation genes in moLCs, as shown for epidermal LCs (Carpentier et al., 2016), but this potential needs to be further evaluated in future studies. Their functional properties render LCs as promising targets for immunotherapy (Deckers et al., 2018). An attractive approach is targeting LCs via their specific C-type lectin receptor Langerin (Stoitzner et al., 2010). Antigen delivery can be achieved by conjugation to anti-Langerin antibodies that are internalized into LCs (Stoitzner et al., 2014), and antigens can be presented to CD4^+^ and CD8^+^ T cells in immunogenic and tolerogenic ways (Flacher et al., 2014). A recent proof-of-principle study delivered a cytotoxic drug via a glycomimetic Langerin ligand to a proliferating Langerin-expressing cell line, which, as a consequence, died by apoptosis (Wamhoff et al., 2019), a strategy for the treatment of LC histiocytosis. Our study highlights that moLCs can be used as an alternative to skin LCs for testing LC-based immunotherapy. Just like skin LCs (Wamhoff et al., 2019), moLCs rapidly bound and internalized Langerin ligand‒coated liposomes. Thus, encapsulated antigens coformulated with adjuvant could be efficiently targeted to moLCs and would allow screening of potential future vaccines for various diseases. Besides Langerin, moLCs also coexpress DEC-205 and DC-SIGN, which extends the range of possible targeting receptors and combinations thereof to determine the efficacy of targeted vaccines.

Altogether, we demonstrate that in vitro‒generated moLCs with GM-CSF, TGF-β1, and the Notch ligand DLL4 closely resembles their in vivo skin counterparts in terms of marker expression, the formation of Birbeck granules, maturation upon cytokine stimulus, and allostimulatory potential with Th1 and Th17 cytokine secretion. They are not only a useful, easily accessible tool to study different functions of LCs but may also help to elucidate their potential for immunotherapies.

## Materials and Methods

### Monocyte isolation

Anonymized peripheral blood from healthy donors was obtained from the local blood bank according to the guidelines by written informed consent and approved by the local ethics committee (Nr. 1265/2019). PBMCs were separated by density gradient centrifugation on Lymphoprep (1.077 g/ml; STEMCELL Technologies, Vancouver, Canada). CD14^+^ monocytes were then isolated by positive magnetic separation using anti-human CD14-magnetic particles (BD Biosciences, Franklin Lakes, NJ) according to manufacturer’s instructions, yielding a purity of more than 95%. The CD14^−^ cell fraction was frozen and used as a source for responder T cells in MLRs.

### MoLC differentiation

Stromal OP9 cells expressing the Notch ligand DLL4 (OP9-DLL4) were provided by Juan Carlos Zúñiga-Pflücker (Sunnybrook Research Institute, Department of Immunology, University of Toronto, Ontario, Canada). Cells were cultured in RPMI1640 (Lonza, Basel, Switzerland) supplemented with 10% heat-inactivated fetal calf serum (PAN-Biotech, Aidenbach, Germany), 2 mM L-glutamine (Thermo Fisher Scientific, Waltham, MA), and 50 U/ml penicillin and 50 μg/ml streptomycin (Thermo Fisher Scientific). Cells were confirmed to be mycoplasma-free by Venor GeM Classics Mycoplasma PCR detection kit (BioProducts, Stockerau, Austria) and tested by flow cytometry for surface DLL4. For the generation of moLCs, 5,000 OP9-DLL4 cells per well were seeded in a 96-well round-bottom plate (Corning, Corning, NY). After 24 hours, 20,000 monocytes per well were added with 50 ng/ml GM-CSF (Sanofi, Paris, France) and 10 ng/ml TGF-β1 (PeproTech, Rocky Hill, NJ), followed by incubation for 3 days at 37 °C. Thereafter, cells were either harvested or cultured for additional 24–48 hours in the presence of a DC-cytokine maturation cocktail consisting of 10 ng/ml IL-1β, 10 ng/ml TNF-α, 10 ng/ml IL-6, and 1 μg/ml prostaglandin E2 (all from PeproTech) or for 24 hours in the presence of 5 μg/ml CpG (Miltenyi Biotec, Bergisch Gladbach, Germany), 20 μg/ml PolyI:C (Sigma-Aldrich, St. Louis, MO), or 100 ng/ml lipopolysaccharide (Sigma-Aldrich).

### Flow cytometry

Flow cytometry analysis was performed on a FACSCanto II and FlowJo software (both from BD Biosciences). Dead cells were excluded using the fixable viability-dye-eFluor780 (Thermo Fisher Scientific), and nonspecific FcR-mediated staining was blocked with blocking reagent (Miltenyi). Stainings were performed for 15 minutes at 4 °C with fluorophore-labeled antibodies as listed in Supplementary Table S1 (Supplementary Material). Fluorescence minus one or isotype-matched antibodies were used as controls. For intracellular staining, Cytofix/Cytoperm kit (BD Biosciences) was used according to the manufacturer’s instructions.

### Electron microscopy

MoLCs were fixed according to standard protocols in 2% glutaraldehyde, and then pelleted, dehydrated, and fixed in resin (all from TAAB Laboratory, Aldermaston, United Kingdom). Ultrathin sections were cut with a diamond knife on an RMC MT-XL ultramicrotome (RMC Boeckeler, Tucson, AZ) and examined with a Philips CM100-Compustage (FEI) Transmission Electron Microscope (Philips, Amsterdam, Netherlands). Images were collected with an AMT-CCD camera (Deben, Bury St Edmunds, UK).

### RNA-seq

Human CD14^+^ monocytes, CD1a^+^Langerin^+^ migratory skin LCs, and moLCs were sorted into 10 μl SMART-Seq v4 lysis buffer on a FACSAria Fusion (BD Biosciences). The SMART-Seq v4 protocol was used for cDNA synthesis. Sequencing libraries were prepared with the Nextera XT library prep kit (Illumina, San Diego, CA). The Illumina NextSeq 500 platform (Illumina) was employed to generate 75bp single-end reads. Subsequent analysis of RNA-seq reads was performed as described in the Supplementary Material.

### MoLC–T cell assay

For the allogeneic MLRs, CD1a^+^Langerin^+^ and CD1a^+^Langerin^−^ cells were sorted on the basis of CD1a and Langerin expression with a FACS Aria II (BD Biosciences). Cells were cultured in the presence or absence of the cytokine maturation cocktail for 24 hours, both supplemented with 50 ng/ml GM-CSF (Sanofi), followed by coculturing with 0.4 μM carboxyfluorescein succinimidyl ester‒labeled (Thermo Fisher Scientific) allogeneic CD14^−^ peripheral blood lymphocytes in the ratios of 1:5, 1:10, and 1:20 for 5 days. On day 5, supernatants from MLRs were collected, and cells were harvested to determine T cell proliferation by carboxyfluorescein succinimidyl ester dilution by flow cytometry. Supernatants were used for a ProcartaPlex Immunoassay (Thermo Fisher Scientific) for measurements of the cytokines IL-2, IL-4, IL-5, IL-9, IL-10, IL-13, IL-17A, and IFN-γ.

### Cytokine detection with ELISA

A total of 100,000 sorted CD1a^+^Langerin^+^ cells were cultured in the presence or absence of 50,000 CD40L cells, 20 μg/ml PolyI:C, or 100 ng/ml lipopolysaccharide in 200 μl medium supplemented with 50 ng/ml GM-CSF. After 24 hours, supernatants were collected and used for IL-12p70 and TNF-α OptEIA ELISA (BD Biosciences). CD40L cells (P3xTBA7, a murine myeloma cell line expressing the human CD40L) were a kind gift from R.A. Kroczek (Berlin, Germany).

### MoLC loading with liposomes

Formulation of AlexaFluor-647 (A647)-labeled liposomes coated with a synthetic glycomimetic ligand for human Langerin were recently described (Wamhoff et al., 2019). Liposomes were incubated with mixed cultures of moLCs and CD1a^+^Langerin^−^ cells at a lipid concentration of 16 μM in RPMI1640 (Lonza) supplemented with 10% fetal calf serum (PAN-Biotech), 2 mM L-glutamine (Thermo Fisher Scientific), 50 U/ml penicillin, and 50 μg/ml streptomycin (Thermo Fisher Scientific) for 1 hour at 37 °C. Ligand binding to Langerin is calcium ion‒dependent and was abrogated by the addition of 10 mM EDTA (Lonza) after 1-hour incubation. Cells were analyzed for A647 signal by flow cytometry.

### Operetta microscopy

Mixed culture of moLCs and CD1a^+^Langerin^−^ cells (50,000 cells per well) were seeded in CellCarrier Ultra plates (PerkinElmer, Waltham, MA) supplemented with 50 ng/ml GM-CSF. They were stained with phycoerythrin-conjugated Langerin (clone MB22-9F5, Miltenyi), Hoechst33342 fluorescence dye (Thermo Fischer Scientific), and 1 μM lysotracker (Thermo Fischer Scientific). A647-labeled Langerin ligand‒targeted or non-targeted liposomes were added for imaging at a lipid concentration of 16 μM. Live cell imaging was performed with a ×63 lens, and pictures were acquired every 5 minutes over 1 hour at 37 °C on the Operetta High-Content Imaging System (PerkinElmer). Samples were analyzed using the Operetta Harmony Software (PerkinElmer).

### Studies that were performed

1. Characterization of moLCs, alloMLR, ELISA, and liposome binding assays was performed at the Department of Dermatology, Venereology & Allergology, Medical University of Innsbruck, Innsbruck, Austria.
2. Electron microscopy and RNA-seq were performed at the Translational and Clinical Research Institute, Faculty of Medical Sciences, Newcastle University, Newcastle upon Tyne, United Kingdom.
3. ProcartaPlex was performed at the Institute of Cell Genetics, Department for Genetics and Pharmacology, Medical University of Innsbruck, Innsbruck, Austria.
4. Operetta Live Cell Imaging was performed at the Institute of Hygiene and Medical Microbiology, Medical University of Innsbruck, Innsbruck, Austria.
5. Liposome formulation was performed at the Department of Biomolecular Systems, Max Planck Institute of Colloids and Interfaces, Potsdam, Germany.
6. Human skin collection was performed at the Department of Plastic, Reconstructive and Aesthetic Surgery, Medical University of Innsbruck, Innsbruck, Austria and at the Translational and Clinical Research Institute, Faculty of Medical Sciences, Newcastle University, Newcastle upon Tyne, United Kingdom.

### Statistical analysis

Data sets in Figure 4 were tested for normality using D’Agostino and Pearson normality test from GraphPad Prism software (GraphPad, San Diego, CA). Paired *t*-test (parametric) or Wilcoxon matched--pairs signed signed-rank test (nonparametric) were used. A *P*-value of ≤ 0.05 was considered statistically significant (∗).

### Data availability statement

The accession number for the RNA-seq data is National Center for Biotechnology Information Gene Expression Omnibus GSE141048.

## ORCIDs

Lydia Bellmann: http://orcid.org/0000-0002-1891-5319

Claudia Zelle-Rieser: http://orcid.org/0000-0002-0752-1795

Paul Milne: http://orcid.org/0000-0002-8278-0463

Anastasia Resteu: http://orcid.org/0000-0002-3783-8806

Christoph H. Tripp: http://orcid.org/0000-0001-5502-7241

Natascha Hermann-Kleiter: http://orcid.org/0000-0003-4389-9813

Viktoria Zaderer: http://orcid.org/0000-0002-0507-0513

Doris Wilflingseder: http://orcid.org/0000-0002-5888-5118

Paul Hörtnagl: http://orcid.org/0000-0001-9183-3320

Maria Theochari: http://orcid.org/0000-0001-8107-5108

Jessica Schulze: http://orcid.org/0000-0002-5570-2128

Mareike Rentzsch: http://orcid.org/0000-0003-2308-8888

Barbara Del Frari: http://orcid.org/0000-0002-3089-7109

Matthew Collin: http://orcid.org/0000-0001-6585-9586

Christoph Rademacher: http://orcid.org/0000-0001-7082-7239

Nikolaus Romani: http://orcid.org/0000-0003-1614-9128

Patrizia Stoitzner: http://orcid.org/0000-0002-8488-6704

## Conflict of Interest

JS and CR declare the filing of a patent covering the use of glycomimetic Langerin ligands for targeting Langerin-expressing cells.

## Figures and Tables

**Supplementary Table S1:** Fluorophore-Labeled Antibodies Used for Flow Cytometry

Antibodies	Clone	Company
CCR7	150503	R&D Systems
CD1a	HI149	BioLegend
CD4	SK3	BioLegend
CD8	RPA-T8	BioLegend
CD14	HCD14	BioLegend
CD40L	24-21	BioLegend
CD45	HI30	BioLegend
CD80	L307.4	BD Biosciences
CD83	HB15e	BioLegend
CD86	IT2.2	BD Biosciences
DC-SIGN	DCN46	BD Biosciences
DEC-205	HD30	BioLegend
DLL4	HMD4-1	BioLegend
HLA-DR	L242	BioLegend
Langerin	MB22-9F5	Miltenyi

## Supplementary Materials and Methods

### LC preparation

Healthy human skin samples were collected after written informed consent and approval by the local ethics committee of the Medical University of Innsbruck, Innsbruck, Austria (AN5003 403/5.10). Whole-skin cell suspensions were prepared at 37 °C by overnight incubation of skin pieces in RPMI1640 (Lonza, Basel, Switzerland) supplemented with 10% heat-inactivated fetal calf serum (PAN-Biotech, Aidenbach, Germany), 2 mM L-glutamine (Thermo Fisher Scientific, Waltham, MA), and 50 μg/ml gentamicin (Thermo Fisher Scientific) containing 1 mg/ml collagenase IV (Worthington Biochemical Corporation, Lakewood, NJ). For RNA sequencing, healthy human skin was obtained from mammoplasty and breast reconstruction surgery after written informed consent and with ethical approval from the Newcastle and North Tyneside Research Ethics Committee. Langerhans cell (LCs) emigrated from epidermal sections during incubation at 37 °C for 72 hours in RPMI1640 (Lonza), supplemented with 10% heat-inactivated fetal calf serum, 2 mM L-Glutamine, 1% Penicillin-Streptomycin (all from PAA Cell Culture Company, Cambridge, United Kingdom), and 50 ng/ml GM-CSF (R&D Biosystems, Minneapolis, MN).

### Analysis of RNA-sequencing data

Reads were trimmed on the basis of quality with Trimmomatic, version 0.36 (Bolger et al., 2014). Bases with quality scores below Q10 (inferred base cell accuracy below 90%) were trimmed, and reads shorter than 50 base pair were dropped. The remaining reads were aligned in the STAR mapping algorithm, version 2.4.0 (Dobin and Gingeras, 2015) to the human reference genome, version GRCh38.p7 (GENCODE release 25). The files were converted from SAM format to the more compressed BAM format with SAMtools, version 1.3 (Li et al., 2009). The count tables were obtained using HTSeq, version 0.6.1 (Anders et al., 2015). ENSEMBL IDs were converted to HGNC symbols using biomaRt, version 2.30.0 (Durinck et al., 2005). Further analysis of the data was undertaken in R, version 3.6.0, and Rstudio, version 1.0.143. Protein coding genes with over 10 reads in total were retained for the analysis. The DESeq2 package, version 1.24.0 (Love et al., 2014) was used for normalization and differential gene expression analysis.

Differentially expressed genes (adjusted *P*-value < 0.05 and fold change over 1.5) for monocyte versus monocyte-derived LC (moLC) and skin LC versus moLC comparisons were split into genes with higher expression in monocytes (2625/4301) and higher expression in moLCs (1676/4301) and genes with higher expression in LC (1073/1801) and higher expression in moLCs (728/1801), respectively. The lists of differentially expressed genes were converted from HGNC symbols to Entrez Gene IDs with biomaRt, version 2.30.0 (Durinck et al., 2005) and used as input for the Gene Ontology analysis performed with the clusterprofiler R package (Yu et al., 2012).

Heatmaps showing the expression of LC-related and TLR and RLR genes were constructed with gplots, version 3.0.1.1. A pseudo count of 1 was added to the DESeq2-normalized expression values, which were then log_2_ transformed and displayed as row z scores.

Geneset enrichment analysis was performed with the GSEA software, version 4.0.2 (Mootha et al., 2003, Subramanian et al., 2005) using the Reactome C-type lectin receptors pathway (R-HSA-5621481) and Gene Ontology terms for antigen presentation in the context of major histocompatibility complex class I (Gene Ontology:0002474) and MHC II (Gene Ontology:0002504). DESeq2-normalized expression values were used as input.

The accession number for the RNA-sequencing data is National Center for Biotechnology Information Gene Expression Omnibus GSE141048.
